# Luteinizing Hormone Surge-Induced *Krüppel-like Factor 4* Inhibits *Cyp17A1* Expression in Preovulatory Granulosa Cells

**DOI:** 10.3390/biomedicines12010071

**Published:** 2023-12-27

**Authors:** Yuri Choi, Okto Lee, Kiyoung Ryu, Jaesook Roh

**Affiliations:** 1 Laboratory of Reproductive Endocrinology, Department of Anatomy & Cell Biology, College of Medicine, Hanyang University, Seoul 04763, Republic of Korea; mini87@hanyang.ac.kr (Y.C.); dhrxh1234@hanyang.ac.kr (O.L.); 2 Department of Obstetrics & Gynecology, College of Medicine, Hanyang University, Guri-si 11923, Republic of Korea; drryuky@hanyang.ac.kr

**Keywords:** *Klf4*, *Cyp17A1*, LH, granulosa cells, luteinization

## Abstract

Previous in vivo and in vitro studies have demonstrated a dramatic up-regulation of *Krüppel-like factor 4* (*Klf4*) in rat preovulatory granulosa cells (GCs) after LH/hCG treatment and its role in regulating *Cyp19A1* expression during the luteal shift in steroidogenesis. In this study, we examined whether *Klf4* also mediates the LH-induced repression of *Cyp17A1* expression in primary rat preovulatory GCs. In response to LH treatment of GCs in vitro, *Cyp17A1* expression declined to less than half of its initial value by 1 h, remaining low for 24 h of culture. Overexpression of *Klf4* decreased basal and *Sf1*-induced *Cyp17A1* expressions and increased progesterone secretion. Reduction of endogenous *Klf4* by siRNA elevated basal *Cyp17A1* expression but did not affect LH-stimulated progesterone production. Overexpression of *Klf4* also significantly attenuated *Sf1*-induced *Cyp17A1* promoter activity. On the other hand, mutation of the conserved Sp1/Klf binding motif in the promoter revealed that this motif is not required for *Klf4*-mediated repression. Taken together, these data indicate that the *Cyp17A1* gene may be one of the downstream targets of *Klf4*, which is induced by LH in preovulatory GCs. This information may help in identifying potential targets for preventing the molecular changes occurring in hyperandrogenic disorders.

## 1. Introduction

Preovulatory granulosa cells (GCs) are characterized by a high content of steroidogenic enzymes and the acquisition of luteinizing hormone (LH) receptors [1]. During the LH surge, GCs undergo molecular and morphological changes, leading to terminal differentiation into luteal cells [2]. These changes are mediated by the coordinated regulation of genes encoding various cell cycle modulators and steroidogenic factors in preovulatory follicles and drive the greater transformation seen in GCs than in theca cells (TCs) as they undergo luteinization [3,4]. As a consequence of luteinization, GCs develop the enzymatic machinery to synthesize progesterone [4,5,6]. A major factor in this steroidogenic shift is a dramatic reduction in the expression of *Cyp19A1* [7,8] along with the acquisition of progesterone-producing steroidogenic enzymes such as *Cyp11A1* and *3βHSD* [9]. GCs start synthesizing progesterone at a greater rate than TCs [10], indicating that they make a major contribution to the marked increase in progesterone production after the LH surge in the corpus luteum (CL).

On the other hand, *Cyp17A1* plays a role in converting C21 precursors (progesterone) to androgens. *Cyp17A1* transcripts also decline to very low or even undetectable levels during the LH surge in the CL [11,12], which may contribute to the accumulation of its precursor, progesterone. Although the expression of *Cyp17A1* is reported to be restricted to TCs [11], it has also been noted in preovulatory GCs [13,14] and even increases slightly in rat GCs after exposure to pregnant mare serum gonadotropin [13]. As it does in TCs, the LH surge reduces *Cyp17A1* to low levels in bovine and human preovulatory GCs [3,14].

Transcriptional regulation of *Cyp17A1* in TCs is well studied [11,15]. In bovine TCs, *Cyp17A1* activity is repressed in response to LH, via the protein kinase A signaling pathway [16]. However, the mechanism of further reduction in *Cyp17A1* after the LH surge, during the reprogramming of GCs to luteal cells, has not been elucidated.

*Krüppel-like factor 4* (*Klf4*) is a zinc finger transcription factor involved in the terminal differentiation of various types of epithelial cells [17]. It is highly expressed in preovulatory GCs after LH/hCG treatment and plays a role in the luteal transition of steroidogenesis by mediating LH-induced repression of *Cyp19A1* [18,19]. Also, clinical investigations point to dysregulation of *Klf4* and *Cyp17A1* expression in the ovaries of patients with polycystic ovary syndrome (PCOS) [20,21]. Based on these findings, it seemed likely that *Klf4* played a role in regulating the transcription of the *Cyp17A1* gene in luteinizing GCs.

Therefore, the present study was undertaken to examine the expression of *Cyp17A1* in cultured GCs in response to LH and to investigate whether the induction of *Klf4* plays a role in regulating *Cyp17A1* expression and progesterone levels. In this work, we used preovulatory GCs isolated from pregnant mare serum gonadotropin-primed immature rat ovaries, which are well-established models of preovulatory GCs.

## 2. Materials and Methods

### 2.1. Animals and Reagents

Immature female Sprague Dawley rats (21 days of age) were purchased from Samtako Biokorea (Osan, Republic of Korea) and housed under controlled temperature, humidity, and light conditions (22–24 °C, humidity 40–50%, 12 h light–dark cycle), with free access to standard rat chow and water. The rats were allowed to acclimate until 26 days of age before being used in experiments. Animal care was consistent with institutional guidelines, and the Hanyang University ACUC committee approved all procedures involving animals (HY-IACUC-2021-0067). Ovine LH (NIH-LH-23) was obtained from the National Hormone and Pituitary Distribution Program (Baltimore, MD, USA). Pregnant mare serum gonadotropin (G4527) was from Sigma–Aldrich (St. Louis, MO, USA). Culture media (McCoy’s 5a medium, Leibovitz L-15 medium), fetal bovine serum (FBS), L-glutamine, and penicillin–streptomycin were purchased from Invitrogen Life Technologies (Carlsbad, CA, USA).

### 2.2. Preparation of Granulosa Cells

In order to induce growth of multiple preovulatory follicles, pregnant mare serum gonadotropin (PMSG) (10 IU) was injected intraperitoneally into immature rats (26 days of age, body weight 55–60 g). Ovaries were dissected 48 h after injection of the PMSG, and preovulatory follicles were punctured in L-15 Leibovitz medium to obtain GCs. Ovarian debris and small follicles were removed, and the remaining medium containing GCs was collected after low-speed centrifugation (500× *g*) for 10 min. The GCs were washed twice with PBS, and suspended in the culture medium (McCoy’s 5a supplemented with 100 U/mL penicillin, 100 μg/mL streptomycin, and 2 mM L-glutamine).

### 2.3. Plasmid Constructs

pCMV3 × FLAG-*Klf4* (FLAG-*Klf4*) was made by subcloning full-length rat *Klf4* cDNA (kindly provided by Prof. H. Kook; Chonnam National University Medical School, Kwangju, Republic of Korea). The plasmid encoding a *Cyp17A1* promoter construct (containing the −2860/+40 region of the human promoter) and steroidogenic factor-1 (*Sf1*) were generous gifts from K. Parker (University of Texas Southwestern Medical Center, Dallas, TX, USA).

The QuickChange site-directed mutagenesis kit (Stratagene, La Jolla, CA, USA) was used to generate the Cyp17A1 promoter constructs with mutated CACCC motifs (−1252/−1241).

Using Stratagene’s web-based QuickChange primer design program, the primers were designed as follows; 5′-GTGTTACAACGAATTCTCAAAAGATCAAGAG ATT-3′ (sense) and 5′-ACAGCAGAGAACCGGTCTGACCACAAATTTACCAGGGCGGAGTTTTTatCttCCCTAGTAAGCCTGAGG-3′ (antisense). The mutant constructs are designated as follows; (ΔKLF/luc) 5′-TAGGGTGGGGAA-3′→5′-TAGGGaaGatAA-3′. Methylated parental DNA templates were digested with DpnI at 37 °C for 1 h and purified. Mutations were confirmed by direct sequencing. The promoterless luciferase reporter plasmid, pGL3-Basic vector, was purchased from Promega (Madison, WI, USA).

### 2.4. Transient Transfection of Granulosa Cells

GCs were prepared as described above and resuspended in electroporation buffer (MPK1025; Thermo Fisher Scientific, Waltham, MA, USA) and then mixed with siRNA or plasmids as indicated in figure legends. Electroporation was performed using a Neon™ Transfection System (MPK5000, Thermo Fisher Scientific, Waltham, MA, USA) and the choice of conditions was a single pulse of 1000 V, 40 ms based on previous studies [18,19]. Transfected cells (1 × 10^5^ cells/well) were resuspended in culture medium and plated in 24-well culture plates. After 6 h of transfection, the medium was replaced and then cells were cultured for 24–36 h. At the end of the culture, the cells were harvested and frozen for total RNA/protein extraction or immediately used for luciferase assays. Conditioned culture media were collected and stored at −20 °C for measurement of progesterone levels. To knockdown *Klf4* with small interfering RNA (siRNA), silence-selected pre-designed and validated siRNA targeting *Klf4* (*Klf4*-siRNA) was purchased from Genepharma (GenePharma Co., Ltd., Shanghai, China); the sequences of the 25-nucleotide sense and antisense RNAs were 5′-CCAUUAUCAAGAGCUCAUGCCACCG-3′ (sense) and 5′-CGGUGGCAUGAGCUCUUGAUAAUGG-3′ (antisense) (accession no: NM_053713). Non-targeting control siRNA (Universal Scrambled siRNA) was also obtained from Genepharma. All experiments were performed at least three times with measurements of duplicate cultures.

### 2.5. Real-Time Quantitative PCR

An RNeasy extraction kit (Qiagen Inc., Valencia, CA, USA) was used to extract total RNA. Total RNA (1 µg) was annealed (5 min at 70 °C) to oligo(dT)18 primers and then reverse transcribed using cDNA synthesis platinum master mix (GenDEPOT, Katy, TX, USA). The Primer-BLAST program (NCBI, Bethesda, MD, USA) was used to design the primers as follows: *Klf4* forward, 5′-GAGAGGAACTCTCTCACATGAAGC-3′ and reverse, 5′-AAGGATAAAGTCTAGGTCCAGGAGA-3′ (NM_053713.1); *Cyp17A1* forward, 5′-CAAGGCTAACGTTGACTCCAG-3′ and reverse, 5′-TGGGTGTAATGAGATGGCTTC-3′ (NM_012753.2). Amplified *18S ribosomal RNA* (*18S rRNA*) (forward, 5′-GCAATTATTCCCCATGAACG-3′ and reverse, 5′-GGCCTCACTAAACCATCCAA-3′) (NM_001025002.1) was used to normalize each reaction (amplification product sizes 185, 167, and 123 bp for *Klf4*, *Cyp17A1*, and *18S rRNA*, respectively). Real-time PCR reactions were carried out in total volumes of 20 μL with Prime Q-Master Mix (with SYBR Green I) (GeNet Bio Inc., Daejeon, Republic of Korea) using a LightCycler 480 II System (Roche Molecular Diagnostics, Indianapolis, IN, USA). PCR conditions were 10 min at 95 °C, 45 cycles of 95 °C for 10 s, 58~60 °C for 10 s, and 72 °C for 10 s. All reactions were run in triplicate (Roche) and relative amounts of the transcript were calculated by comparing mean values with the control values. Data are expressed as means ± standard deviations (SDs) of triplicate measurements in three independent experiments.

### 2.6. Western Blot Analysis

GCs grown as described above were collected and washed with cold PBS before lysis in Laemmli buffer containing β-mercaptoethanol, and the resulting cell lysate was boiled for 3 min to denature proteins. Samples of 30 μg were loaded per lane and resolved by 8% SDS-PAGE gel electrophoresis, and proteins were transferred onto nitrocellulose membranes (Amersham Pharmacia Biotech, Arlington Heights, IL, USA). Membranes were blocked for 2 h at room temperature (RT) in TBS-0.1% Tween containing 5% fat-free dry milk, and incubated at 4 °C overnight with anti-KLF4 antibody (abx006830, Abbexa Ltd., Cambridge, UK) or anti-CYP17A1 antibody (MBS820246, MyBioScience, Inc., San Diego, CA, USA) diluted 1:1000 and 1:500, respectively, in TBS-0.1% Tween solution. The membranes were washed with TBS-0.1% Tween and blotted with peroxidase-conjugated donkey anti-rabbit secondary antibody (1:8000) (Boehringer Mannheim, Indianapolis, IN, USA) for 2 h. Immunolabeled proteins were detected with an enhanced chemiluminescence kit (Amersham Pharmacia Biotech., Little Chalfont, UK). The 55 kDa KLF4 and 50 kDa CYP17A1 proteins are indicated in the figures. To ensure that lysates were loaded equally, the blots were stripped and incubated with β-actin (1:3000) (ab8227, Abcam, Waltham, MA, USA).

### 2.7. Assessment of Progesterone Production

GCs (1 × 10^5^ cells/well) were transfected with *Klf4* expression plasmid (0.1 and 0.3 μg/well) or *Klf4* siRNA (200 nM/well). After 6 h, they were transferred to LH (200 ng/mL) or control medium for 24–36 h, and conditioned media were harvested for the hormone assay. Progesterone levels were measured using an enzyme-linked immunosorbent assay (ELISA) kit (CSB-E07282r, Cusabio Biotech Co., Ltd., Wuhan, China) following the manufacturer’s protocol. Intra- and inter-assay coefficients of variation were less than 15%, and the limit of detection was 0.8 ng/mL under our conditions. To achieve concentrations within the assay range, the conditioned media were diluted, and serial dilutions were found to behave in a linear fashion using progesterone standards. Absorbance was read against a blanking well at 450 nm within 15 min in an ELISA Reader (BioRad, Hercules, CA, USA). All samples were run in duplicate. Data were collected from three independent experiments.

### 2.8. Luciferase Assays

GCs were prepared as described above. Cells (1 × 10^5^ cells/well) were cotransfected with 1.5 μg of *Cyp17A1* promoter luciferase plasmid (wild-type or mutant constructs) or pGL3-Luc, empty vector, and increasing concentrations of plasmids encoding *Klf4* (10, 30, 100 ng/well) and/or *Sf1* (10, 30 ng/well) using a Neon™ Transfection System. In order to correct for differences in transfection efficiency, a Renilla luciferase reporter vector (50 ng/well) (Promega) was co-transfected as an internal control. Transfected GCs plated in 24-well plates were cultured for 24–36 h. To harvest cells, cells were washed with PBS, Reporter Lysis Buffer (100 μL) (Promega Corp., Madison, WI, USA) was added into each well, and 20 μL of the supernatant was used for the luciferase assay using a luminometer (FB12, Berthold Technologies, Bad Wildbad, Germany). Firefly luciferase activities were normalized by Renilla luciferase activities and data are expressed as means ± SD of triplicate measurements in three independent experiments.

### 2.9. Data Analysis

Data are expressed as means with standard deviations (SDs) of at least three independent experiments. IBM SPSS Statistics 26 for Windows (IBM Corp., Armonk, NY, USA) was used for all data analysis. Statistical significant differences were determined using the Kruskal–Wallis test followed by Dunnett’s post hoc test for multiple-group comparisons and the Mann–Whitney U-test for two-group comparisons. *p* < 0.05 was considered significant.

## 3. Results

### 3.1. Effect of LH on Klf4 and Cyp17A1 Expression in Cultured Preovulatory GCs

**Figure 1:**
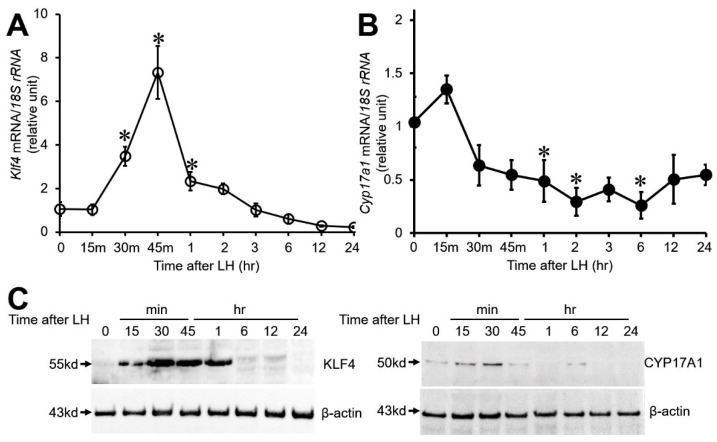
Effect of LH on *Klf4* and *Cyp17A1* mRNA and protein levels in cultured preovulatory GCs. Real-time RT-PCR analysis of (**A**) *Klf4* and (**B**) *Cyp17A1* mRNA levels. GCs obtained from rat preovulatory follicles (as described above) were cultured with LH (200 ng/mL) for the indicated times. *18S rRNA* was used to normalize reactions. Values were calculated as fold changes relative to values at 0 h and are expressed as means ± SDs of three independent preparations of GCs. * *p* < 0.001 vs. 0 h. (**C**) Immunoblot analysis of KLF4 (left panel) and CYP17A1 (right panel). Lysates were immunoblotted with anti-KLF4 antibody (1:1000) (abx006830) or anti-CYP17A1 antibody (1:500) (MBS820246). Arrows indicate bands corresponding to KLF4 (55 kDa), CYP17A1 (50 kDa), and β-actin (43 kDa).

To characterize the LH-induced regulation of *Klf4* and *Cyp17A1* expression in GCs, preovulatory GCs were treated with a luteinizing dose of LH (200 ng/mL) for the indicated times and collected for real-time PCR and Western blot analysis. As previously demonstrated [18], LH treatment rapidly and transiently increased *Klf4* mRNA (Figure 1A) and protein levels (Figure 1C, upper panel) within 1 h. On the other hand, *Cyp17A1* mRNA levels began to decrease rapidly from 30 min and a marked decrease was noted by 1 h (*p* < 0.001 vs. 0 h). After that, *Cyp17A1* mRNA levels remained low throughout the 24 h culture period (Figure 1B). *Cyp17A1* protein levels also declined very quickly after peaking at 30 min (Figure 1C, right panel).

### 3.2. Effect of Klf4 on Basal and Sf1-Mediated Cyp17A1 Gene Expression in Cultured GCs

**Figure 2:**
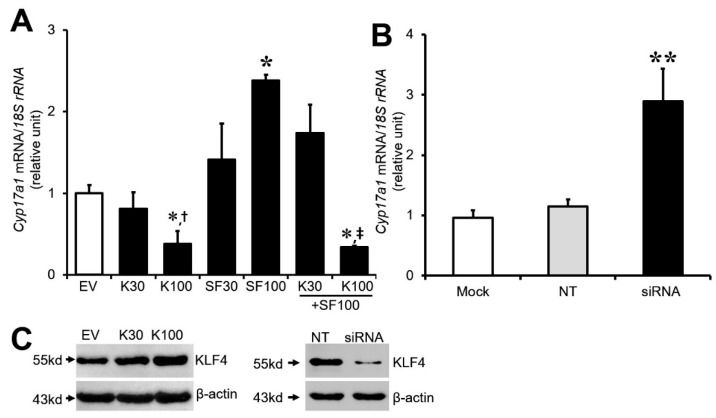
Regulation of *Cyp17A1* expression by *Klf4* in preovulatory granulosa cells. GCs were obtained from preovulatory follicles of rat ovaries 48 h after injection of pregnant mare serum gonadotropin. (**A**) Real-time RT-PCR analysis of *Cyp17A1* mRNA levels in GCs cotransfected with increasing amounts of *Klf4* plasmid (30, 100 ng/well), *Sf1* plasmid (30, 100 ng/well), or empty vector (EV). EV, empty vector; SF30, *Sf1* 30 ng/well; *SF1*00, *Sf1* 100 ng/well; K30, *Klf4* 30 ng/well; K100, *Klf4* 100 ng/well. * *p* < 0.05 vs. EV; ^†^ *p* < 0.05 vs. K30; ^‡^ *p* < 0.001 vs. *SF1*00. (**B**) Real-time RT-PCR analysis of *Cyp17A1* mRNA levels in GCs transfected with *Klf4* siRNA (200 nM) or control siRNA (200 nM) (NT). Mock, mock-transfected control; NT, non-target control siRNA; siRNA, *Klf4*-specific siRNA. ** *p* < 0.05 vs. NT. *18S rRNA* was used to normalize reactions. Values were calculated as fold changes relative to controls (EV or NT) and are expressed as means ± SDs of at least three independent experiments. (**C**) Immunoblot analyses of KLF4 protein in lysates of transfected GCs to confirm *Klf4* overexpression (left panel) and knockdown (right panel). Arrows indicate bands corresponding to KLF4 (55 kDa) and β-actin (42 kDa).

*Klf4* has been linked to *Cyp19A1* repression after the LH surge in luteinizing GCs [19]. Furthermore, *Klf4* was most strongly expressed in the GCs of preovulatory follicles as well as granulosa lutein cells of newly formed or mature corpus luteum, although its expression was heterogeneous (Supplemental Figure S1). We therefore hypothesized that the ovarian increase in *Klf4* after the LH surge also participates in the regulation of *Cyp17A1* in preovulatory GCs. To determine whether *Klf4* modulates the expression of *Cyp17A1*, GCs isolated from preovulatory follicles were transiently transfected with a *Klf4* expression plasmid or *Klf4*-specific siRNA. After 24–36 h, *Cyp17A1* mRNA levels were quantified by real-time RT-PCR. Overexpression of and reduction in *Klf4*, respectively, were confirmed by immunoblot analysis (Figure 2C). Overexpression of *Klf4* led to a significant reduction in the basal level of *Cyp17A1* mRNA, which was about 0.4-fold of the control at 100 ng of *Klf4* plasmid (*p* < 0.01), and *Sf1*-stimulated expression was also completely abolished in the cells receiving 100 ng *Klf4* expression plasmid (Figure 2A). Conversely, the knockdown of *Klf4* expression with siRNA significantly increased basal *Cyp17A1* mRNA compared with scrambled siRNA-transfected cells (2.89-fold) (*p* < 0.05) (Figure 2B). These data demonstrate an inhibitory effect of *Klf4* on *Cyp17A1* expression.

### 3.3. Effect of Klf4 on Basal and LH-Stimulated Progesterone Production in Cultured GCs

**Figure 3:**
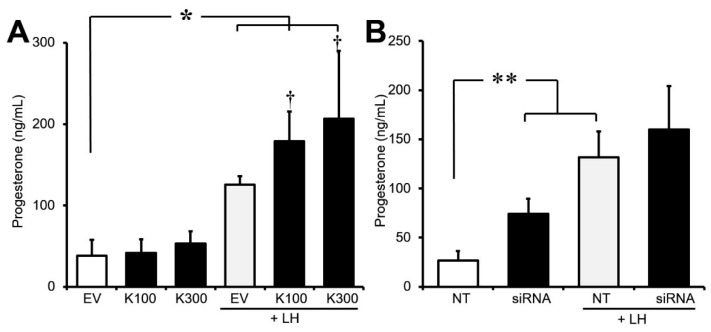
Effects of *Klf4* overexpression and knockdown on basal and LH-stimulated progesterone production by cultured granulosa cells. GCs were obtained from preovulatory follicles of rat ovaries 48 h after injection of pregnant mare serum gonadotropin. (**A**) For overexpression, GCs were transfected with *Klf4* plasmid (100, 300 ng/well) or empty vector (EV), and (**B**) for knockdown, they were transfected with *Klf4* siRNA (200 nM) or control non-target siRNA (NT) and cultured for 24–36 h in the presence or absence of LH (200 ng/mL). At the end of the incubation, supernatant progesterone levels were assayed by ELISA. Values were calculated as fold changes relative to the value for EV-transfected cells or NT-transfected cells and are expressed as means ± SDs of at least three independent experiments. EV, empty vector; *Klf4*, FLAG-*Klf4* plasmid; NT, non-target control siRNA; siRNA, *Klf4*-specific siRNA. * *p* < 0.05 vs. EV; ^†^ *p* < 0.05 vs. EV + LH; ** *p* < 0.05 vs. NT.

Since *Cyp17A1* is known to have the capacity to convert C21 precursors (progesterone) to androgens, *Klf4* may increase progesterone levels by directly downregulating the transcription of *Cyp17A1* in luteinizing GCs. To evaluate whether *Klf4* affected progesterone levels, GCs transfected with the *Klf4* overexpression construct or *Klf4*-specific siRNA were cultured in the absence or presence of LH for 24–36 h, and supernatant progesterone concentrations were measured. *Klf4* overexpression significantly increased LH-stimulated progesterone production (*p* < 0.05 vs. CT) (Figure 3A). Unexpectedly, a reduction in *Klf4* expression with siRNA also significantly enhanced basal progesterone production (2.8-fold), whereas inhibition of *Klf4* expression had no effect on LH-stimulated progesterone levels (Figure 3B).

### 3.4. Regulation of Cyp17A1 Promoter Activity by Klf4

**Figure 4:**
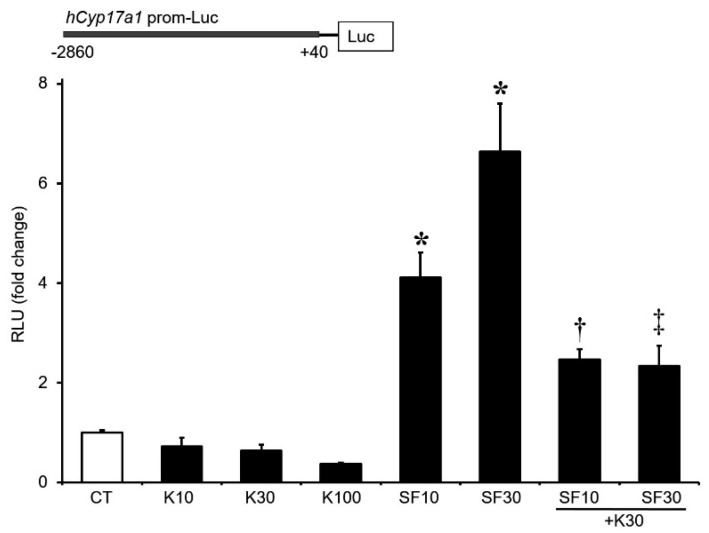
*Klf4* inhibition of basal and *Sf1*-stimulated *Cyp17A1* promoter activity in cultured granulosa cells. GCs were obtained from preovulatory follicles of rat ovaries 48 h after injection of pregnant mare serum gonadotropin. Cells were co-transfected with a *Cyp17A1* promoter luciferase reporter construct (1.5 μg/well) consisting of the proximal 2900 bp of the promoter with increasing amounts of *Sf1* plasmid (10 and 30 ng/well) with/without *Klf4* plasmid (10, 30, and 100 ng/well), and cultured for 24–36 h. Cell lysates were assayed for luciferase, expressed as relative light units (RLU), and normalized to Renilla luciferase activity in co-transfected cells. Values are fold changes relative to control (CT) and are means ± SDs of three independent experiments, each performed in triplicate. CT, cells transfected with *Cyp17A1* promoter only. * *p* < 0.05 vs. CT; ^†^ *p* < 0.05 vs. *SF1*0 only; ^‡^ *p* < 0.05 vs. SF30 only.

To determine whether *Klf4* acts on the *Cyp17A1* promoter, the *Klf4* expression vector and a *Cyp17A1* promoter construct were cotransfected into cultured GCs. *Klf4* overexpression caused a dose-dependent decrease in basal promoter activity, although the effect did not attain statistical significance. In addition, the *Sf1*-stimulated transcriptional activity of the *Cyp17A1* promoter was significantly attenuated by cotransfection of the *Klf4* expression plasmid (Figure 4). These findings imply that *Cyp17A1* is directly or indirectly affected by LH-induced *Klf4*.

### 3.5. Sp1/Klf4-Binding Sequences Are Not Involved in Klf4 Repression of Cyp17A1

**Figure 5:**
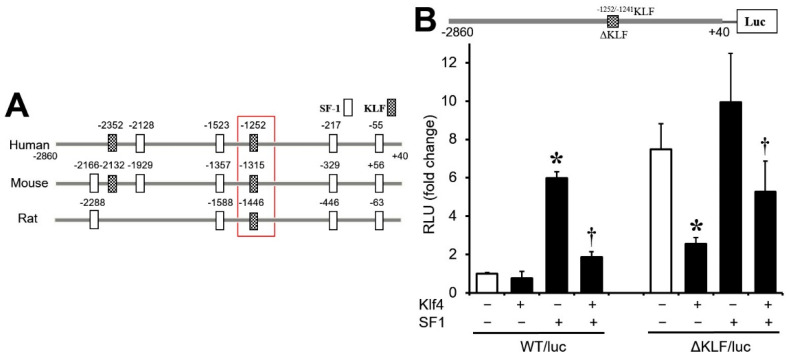
The CACCC motif is not required for *Klf4*-mediated inhibition of *Cyp17A1* promoter activity. (**A**) Schematic representation of the human, rat, and mouse *Cyp17A1* promoter indicating the relative position of the conserved *Klf4* binding site (indicated in red square). *Sf1*, steroidogenic factor 1; KLF, CACCC motif. (**B**) Schematic showing the mutated regions of the *Cyp17A1* promoter (upper panel). *Cyp17A1* promoter constructs (1.5 μg/well) containing the wild-type or mutated CACCC motif were co-transfected into GCs together with *Klf4* plasmid (30 ng/well) or *Sf1* plasmid (30 ng/well). Values were calculated as fold changes relative to control and are expressed as the means ± SDs of three independent experiments, each performed in triplicate. Control (CT, cells transfected with either wild-type or mutated *Cyp17A1* promoter only); WT/luc, wild-type *Cyp17A1* promoter; ΔKLF/luc, promoter with mutation at −1252/−1241KLF site (GTGGGGA→GaaGatA). * *p* < 0.05 vs. CT; ^†^ *p* < 0.05 vs. CT + SF1.

Next, we examined the putative promoter region (~2900 bp upstream of the transcription start site) of human *Cyp17A1* using a web-based transcription factor prediction program. This revealed the presence of three *Sf1*- and one Sp1/*Klf4*-binding motif (CACCC) (−1252/−1241 bp), all of which are conserved in humans, mice, and rats (Figure 5A). To assess the functional importance of the conserved *Klf4*-binding motif in the inhibition of *Cyp17A1* expression by *Klf4*, we mutated the CACCC motif (generating plasmid ∆KLF/luc) (Figure 5B, upper panel) and co-transfected it with the *Klf4* and/or the *Sf1* expression vector into preovulatory GCs. Mutation of the *Klf4* binding site did not alter the inhibitory effect of *Klf4* on basal and *Sf1*-stimulated transcriptional activity (Figure 5B). Unexpectedly, the basal luciferase activity of ΔKLF/luc was markedly enhanced (more than seven-fold) compared with WT/luc. These results indicate that the Sp1/*Klf4*-binding motif (−1252/−1241 bp region) in the human *Cyp17A1* promoter is not responsible for the *Klf4*-mediated repression of *Cyp17A1* promoter activity.

## 4. Discussion

A major focus of this study was to understand the mechanism by which *Cyp17A1* expression is repressed in preovulatory GCs after the LH surge, and we have presented evidence that *Klf4* mediates the LH-induced repression of *Cyp17A1* expression in these cells.

It is well established that the LH surge initiates a shift in GC steroidogenesis. After the surge, luteinized GCs constitute the major component of the corpora lutea and are the main source of ovarian progesterone [3,4,5,6]. The decline in estradiol and rise in progesterone synthesis after the LH surge appear to be related to a decline in androgen biosynthesis and loss of *Cyp17A1* activity [11,12]. *Cyp17A1* has a key enzyme role in androgen synthesis, converting progesterone to androstenedione. Previous studies have shown that *Cyp17A1* expression drops to low levels in the GCs and TCs of preovulatory follicles at the time of luteinization [12,22]. Although *Cyp17A1* expression occurs mainly in TCs [11], weak expression has also been found in GCs [13,14]. Several groups have reported that the LH surge reduces *Cyp17A1* expression to low levels in preovulatory GCs as it does in TCs, but they examined only one time point after LH exposure [3,13,14]. In agreement with these observations, we found that *Cyp17A1* mRNA expression fell to approximately 0.3-fold of its basal level by 2 h of LH treatment and remained low during a further 24 h of culture (Figure 1B). Similarly, *Cyp17A1* protein levels declined very quickly after peaking at 30 min. (Figure 1C).

*Klf4* is one of several transcription factors that are transiently induced via the protein kinase A signaling pathway within 30 min of the LH surge in preovulatory GCs (Figure 1) [18] and strongly expressed in the GCs of preovulatory follicles and luteinized GCs of newly formed CLs in the rat ovary (Supplementary Figure S1). Previous studies have indicated that *Klf4* participates in regulating steroidogenic genes such as *StAR* and *Cyp11A1* in porcine granulosa luteal cells [23] and *Cyp19A1* in rat preovulatory GCs [19]. In the present study, *Klf4* overexpression was shown to down-regulate basal and *Sf1*-stimulated *Cyp17A1* expression in preovulatory GCs, leading to increased C21 precursor and progesterone levels (Figure 3A), whereas knockdown of endogenous *Klf4* expression led to increased *Cyp17A1* expression (Figure 3B), as it did in the case of *Cyp19A1* [19]. On the other hand, inhibition of *Klf4* expression had no effect on LH-stimulated progesterone levels (Figure 3B). These results suggest that a specific level of *Klf4* may be needed for the optimal regulation of progesterone production during the luteal transition of GCs. According to published studies, *Cyp17A1* expression in luteinizing TCs is inhibited as a result of repression of *Sf1* by *c-fos* [24]. Therefore, it will be of interest to determine whether c-fos also contributes to *Cyp17A1* expression in luteinizing GCs.

*Klf4* has been shown to interact with CACCC and GC-rich motifs [17,25], and a conserved CACCC motif was noted in the *Cyp17A1* promoter (Figure 5A). However, alteration of the CACCC sequence did not impair *Klf4*-mediated repression; instead, and unexpectedly, it enhanced the transcriptional activity of a *Cyp17A1* promoter reporter construct in preovulatory GCs (Figure 5B), indicating that this motif is not required for *Klf4*-mediated repression and that other elements must be involved on the CACCC motif.

*Sf1* (Steroidogenic factor-1), an orphan nuclear receptor, is essential for gonadal development [26] and positively regulates the transcription of enzymes involved in ovarian steroidogenesis including *Cyp11A1*, *Cyp19A1*, and *Cyp17A1* by binding to specific sites in their promoter regions [22,27]. In rodents, *Sf1* is widely expressed in the ovary and its expression in preovulatory GCs is modulated by gonadotropins [28]. *Klf4* is able to suppress the well-established *Sf1* induction of pro-proliferative genes and *Cyp19A1* by interfering with the recruitment of *Sf1* or competing with *Sf1* for binding to recognition motifs within the promoter region [19,29]. Although a number of possible mechanisms may be responsible for the inhibition of *Cyp17A1* transcription by *Klf4*, *Klf4* may interact with *Sf1* to competitively inhibit its binding to the *Cyp17A1* promoter as it does in the case of *Cyp19A1* [19]. Furthermore, *Sf1* expression in GCs is rapidly reduced to very low levels in response to the LH surge [30]. Along with this, LH-induced *Klf4* may also inhibit *Sf1* activity, ultimately leading to the sharp reduction in *Cyp17A1* expression that we observed. Although additional studies are required to clarify the exact mechanism controlling the expression of this gene in GCs, these data provide experimental evidence that *Cyp17A1* is one of the downstream targets of *Klf4* in preovulatory GCs. However, this needs to be confirmed in human GCs from preovulatory follicles, as findings in humans may differ from those in experimental animals.

Hyperandrogenism is one of the most prominent clinical features of patients with PCOS [20,21]. Elevated expression of *Cyp17A1* has been reported in the GCs, TCs, and even preovulatory oocytes of PCOS patients [20,21,31], suggesting that gene alterations in GCs and oocytes are responsible for the poor oocyte quality and decreased fertilization rates in IVF patients with PCOS [32]. Furthermore, *Klf4* expression is down-regulated in the ovaries of PCOS patients [20]. In view of the involvement of *Klf4* in *Cyp17A1* expression, down-regulation of *Klf4* may contribute to the increased *Cyp17A1* expression found in PCOS patients.

Increased understanding of the ability of *Klf4* to inhibit *Cyp17A1* production may suggest a target for overcoming the molecular aberrations underlying hyperandrogenic disorders such as PCOS.

## Data Availability

The authors confirm that the data supporting the findings of this study are available within the article and its Supplementary Materials.

## Supplementary Materials

The following supporting information can be downloaded at: https://www.mdpi.com/article/10.3390/biomedicines12010071/s1, Figure S1: Immunohistochemical localization of KLF4 in rat ovaries. Ovaries were sectioned at 0, 2, 24, and 48 h after injection of pregnant mare serum gonadotropin-primed rats with hCG and incubated with KLF4 antibody (NBP2-24749). Positive staining appears as a dark brown precipitate. (A) Representative images of ovaries from rats treated with pregnant mare serum gonadotropin for 48 h. Positive signals were detected in the GCs (arrows) of preovulatory follicles, but not in TCs. (B) Representative images of an ovarian section obtained 2 h after hCG injection. Positive signals were detected in the GCs of follicles, with stronger expression in the cumulus and antral GCs (arrows) of ovulatory follicles. Positive signals were also seen in TCs (arrowheads). Representative images of a section of an ovary obtained (C) 24 h and (D) 48 h after hCG injection. Luteinized GCs of the newly formed corpus luteum (nCL) and mature CL (arrow) stained more intensely than those of TCs. GCs, granulosa cells; TCs, theca cells; SF, secondary follicle; POF, preovulatory follicle; nCL, newly formed corpus luteum; CL, mature corpus luteum. Bar = 100 μm.
