# Supplementary material for: Luteinizing Hormone Surge-Induced Krüppel-like Factor 4 Inhibits Cyp17A1 Expression in Preovulatory Granulosa Cells
Source: Biomedicines. 2023 Dec 27;12(1):71. doi: 10.3390/biomedicines12010071 (PMC10813437; doi:10.3390/biomedicines12010071)
Supplement: Supplementary file 1 [file biomedicines-12-00071-s001.zip › biomedicines-2774406-supplementary.pdf]

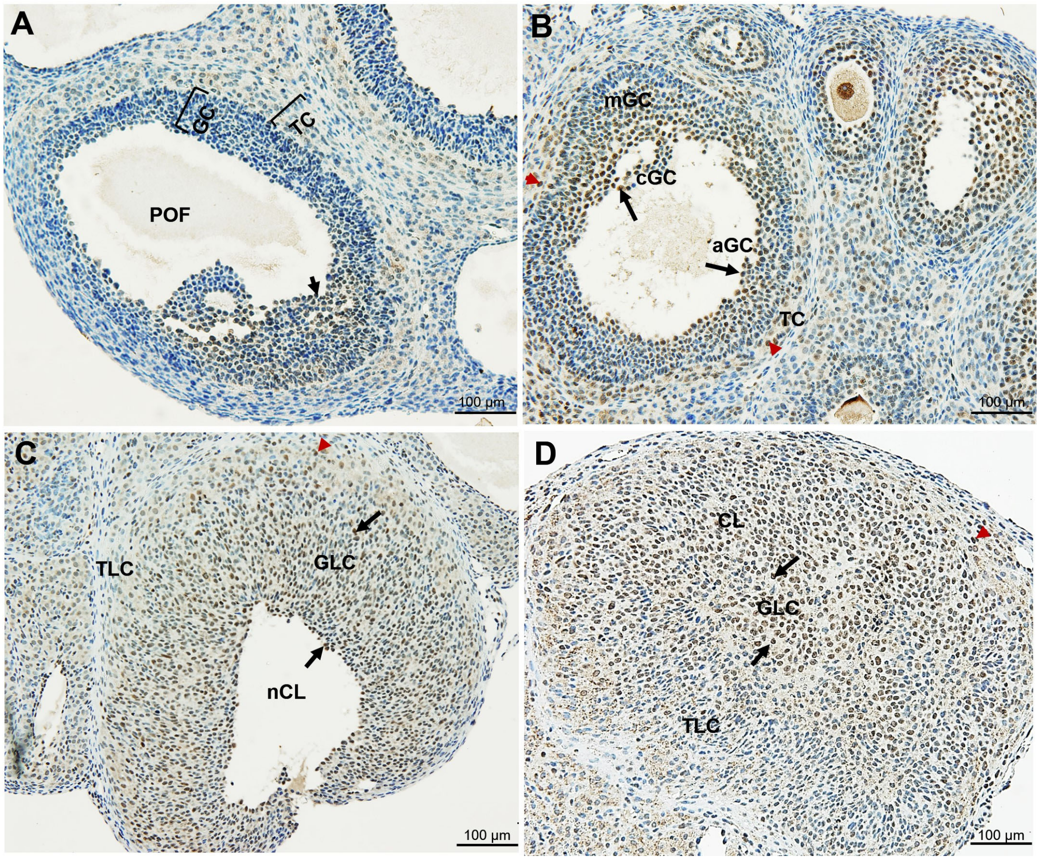

Figure S1. Immunohistochemical localization of KLF4 in rat ovaries. Ovaries were sectioned at 0, 2, 24, and 48 h after injection of pregnant mare serum gonadotropin-primed rats with hCG and incubated with KLF4 antibody (NBP2-24749). Positive staining appears as a dark brown precipitate. (**A**) Representative images of ovaries from rats treated with pregnant mare serum gonadotropin for 48 h. Positive signals were detected in the GCs (arrows) of preovulatory follicles, but not in TCs. (**B**) Representative images of an ovarian section obtained 2 h after hCG injection. Positive signals were detected in the GCs of follicles, with stronger expression in the cumulus and antral GCs (arrows) of ovulatory follicles. Positive signals were also seen in TCs (arrowheads). Representative images of a section of an ovary obtained (**C**) 24 h and (**D**) 48 h after hCG injection. Luteinized GCs of the newly formed corpus luteum (nCL) and mature CL (arrow) stained more intensely than those of TCs. GCs, granulosa cells; TCs, theca cells; SF, secondary follicle; POF, preovulatory follicle; nCL, newly formed corpus luteum; CL, mature corpus luteum. Bar = 100 µm.
